# Neuro Navigation Versus Conventional Spinal Techniques in Analyzing Nerve Injury and Anatomical Accuracy: A Systematic Review

**DOI:** 10.7759/cureus.68760

**Published:** 2024-09-05

**Authors:** Omar A Mahroq, Shakirat Ganiyu, Rithish Nimmagadda, Vemparala Priyatha, Bushra Firdous Shaik, Excel O Ernest-Okonofua, Safeera Khan

**Affiliations:** 1 Orthopedic Surgery, California Institute of Behavioral Neurosciences and Psychology, Fairfield, USA; 2 Internal Medicine, California Institute of Behavioral Neurosciences and Psychology, Fairfield, USA; 3 Family Medicine, California Institute of Behavioral Neurosciences and Psychology, Fairfield, USA

**Keywords:** accuracy, complications, neuronavigation guidance, pedicle screws accuracy, spinal surgery

## Abstract

Neuronavigation, a computer-assisted surgical technique, enhances the accuracy of spinal surgery by using medical imaging to guide the surgeon's instruments. This method mitigates the serious complications of screw misplacement, such as dural tears, nerve damage, vascular injuries, and internal organ damage, by integrating pre-operative imaging data with real-time intraoperative sensor readings. Because of this integration, it is possible to visualize the spine in three dimensions, guaranteeing accurate instrument placement and greatly lowering the risk of complications. Despite its growing popularity, the benefits of neuronavigation in spinal instrumentation are debated. While some studies report improved accuracy in pedicle screw placement, others find no significant difference compared to conventional freehand techniques. Further research is required to determine the long-term benefits of neuronavigation, including its impact on patient outcomes, like reduced pain and improved function. This systematic review will evaluate the evidence on the risks and benefits of neuronavigation in spinal instrumentation surgery, compared to conventional techniques.

## Introduction and background

Neuronavigation is a computer-assisted surgery technique that uses medical imaging to guide the surgeon's instruments during surgery. It is used in various surgical specialties, including neurosurgery, orthopedics, and spine surgery.

Computer navigation has become increasingly popular in spinal surgery because it helps surgeons operate more accurately [1,2]. One way of using minimally invasive spine (MIS) surgery is placing pedicle screws accurately, which requires major changes to the surgical techniques [3].

As surgical techniques and imaging technology improve, MIS surgery is becoming more advanced. While traditional imaging methods like X-rays and fluoroscopy are still common, newer tools, such as three-dimensional fluoroscopy, cone-beam computed tomography (CBCT), and intraoperative CT/MRI, are emerging as game-changers [4,5]. These advancements involve improved imaging technology and advanced surgical techniques. Screw misplacement during spinal surgery can lead to serious complications, including dural tears, nerve damage, vascular injuries, and even damage to internal organs [6,7]. To overcome this drawback, computer navigation has become a useful tool. It does this by tracking the position of the surgeon's instruments using a combination of preoperative imaging data and real-time intraoperative sensor readings. This enables three-dimensional visualization of the spine and accurate instrument placement in relation to the surrounding anatomy, which can greatly improve screw placement accuracy and reduce the risk of complications [8].

This systematic review will evaluate the evidence on the risks and benefits of neuronavigation in spinal instrumentation surgery compared to conventional techniques, providing critical information for patients and surgeons to make informed decisions about the best treatment options.

## Review

Materials and methods

This systematic review was conducted following the Preferred Reporting Items for Systematic Reviews and Meta-Analyses (PRISMA) guidelines [9]. The initial search for relevant studies was conducted across multiple databases and registers, including PubMed, PMC, Medline, Cochrane Library, MDPI, and Science Direct. Various search strategies were used to ensure comprehensive coverage of the literature related to neuronavigation-related injuries during spinal instrumentation. Inclusion and exclusion criteria were established to select relevant articles, focusing on original research articles written in English and reporting on the use of neuronavigation in spine surgery and associated complications or adverse events. The methodological quality of the included studies was assessed using the Joanna Briggs Institute (JBI) and Newcastle-Ottawa Scale. Data extraction was performed by two reviewers independently, using a pre-designed data extraction form, and all data were managed using EndNote software.

These search strategies were executed across various databases to ensure comprehensive coverage of relevant literature about neuronavigation-related injuries during spinal instrumentation. Table 1 shows the search strategy.

**Table 1:** Comparison of search strategies for identifying papers on neuronavigation-related spine injuries

Search strategy	Database used	Number of papers identified
neuronavigation AND instrumentation AND injury	PubMed	35
( "Neuronavigation/adverse effects"[Majr] OR "Neuronavigation/instrumentation"[Majr] ) AND ( "Spine/blood supply"[Majr] OR "Spine/innervation"[Majr] OR "Spine/surgery"[Majr] ) AND ( "Vascular System Injuries/blood"[Majr] OR "Vascular System Injuries/cerebrospinal fluid"[Majr] OR "Vascular System Injuries/complications"[Majr] OR "Vascular System Injuries/surgery"[Majr]	PubMed MESH	801
((Injury) AND (Spine Surgery)) AND (Neuronavigation)	PubMed advanced (Tw)	42

Any disagreements between reviewers during the screening process were resolved through discussion and consensus. Table 2 illustrates the eligibility criteria.

**Table 2:** Studies on neuronavigation for spine surgery

Inclusion criteria	Exclusion criteria
Original research articles	Non-original research (e.g., reviews, case reports, editorials)
Focused on the use of neuronavigation in spine surgery	Published in languages other than English
Reported on patients with spinal injuries	Did not focus on the use of neuronavigation
Included data on complications and/or adverse events	Did not involve patients with spinal injuries
Papers focusing on all age groups	Lacked data on complications and/or adverse events
Papers focusing on neuronavigation including spinal instrumentation	Grey literature
Papers written and published in English	Papers focusing on ICU patients or pregnant women

Data Extraction and Synthesis

Data extraction was performed by two reviewers independently, using a pre-designed data extraction form. The form collected information on study characteristics (e.g., author, year of publication, study design, sample size), neuronavigation system used, type of spinal surgery, type of spinal injury, and reported complications and/or adverse events. Extracted data were then synthesized narratively and presented in tables and figures.

All data were managed using EndNote software. Due to the anticipated heterogeneity in study designs and data reporting, a meta-analysis was not performed. Instead, a qualitative analysis was conducted to provide a comprehensive overview of the current evidence on the use of neuronavigation in spine surgery for patients with spinal injuries, focusing on the reported rates and types of complications and/or adverse events. Table 3 shows how we checked the quality of each paper.

**Table 3:** Cochrane appraisal +, low risk of bias; -, high risk of bias; ?, unclear risk of bias

Study	Random sequence generation	Allocation sequence concealment	Blinding of participants and personnel	Blinding of outcome assessment	Incomplete outcome data	Selective outcome reporting	Other potential sources of bias
Xu et al. (2020) [1]	?	?	?	?	+	+	+
Santoro et al. (2019) [2]	?	?	?	?	+	+	+
Conrads et al. (2023) [8]	?	?	?	?	+	+	+
Lucifero et al. (2021) [10]	?	?	?	?	+	+	+
Wang et al. (2022) [11]	?	?	?	?	+	?	+
Sharma et al. (2022) [12]	?	?	?	?	?	?	?
Verma et al. (2016) [13]	?	?	?	?	?	?	?
Florea et al. (2020) [14]	?	?	?	?	?	?	?
Houten et al. (2017) [15]	?	?	-	-	?	?	?
Yurac et al. (2021) [16]	-	-	?	+	+	?	-
Buchholz et al. (2015) [17]	?	?	?	?	+	+	+
Yu et al. (2019) [18]	?	?	-	-	?	?	-
Hara et al. (2020) [19]	?	?	-	-	?	?	-
Meyer et al. (2020) [20]	?	?	-	-	?	?	-

This systematic review did not require ethical approval, as it was a secondary data analysis of existing published research.

Results

The PRISMA flow diagram outlines the study selection process used in this systematic review [9].

Identification of Studies

The initial search for relevant studies identified 1,318 records. After removing 21 duplicate records and 440 studies marked as ineligible by automation tools, 857 records were screened for eligibility.

Screening

After reviewing the titles and abstracts, 734 records were excluded as they were not relevant to the research topic. This left 123 records for further assessment.

Eligibility Assessment

The full text of these 123 records was thoroughly assessed for eligibility based on predefined criteria. Seventy-one reports were excluded because they were irretrievable. The remaining 52 were assessed for eligibility; 38 were further excluded due to different reasons: not related to the research topic (26), full text not found (nine), and low quality of study design (three).

Included Studies

Finally, 14 studies were deemed eligible for inclusion in the systematic review. These studies covered a range of research designs, including one meta-analysis, three case studies, three case series, one literature review, one retrospective cohort study, two retrospective case-control studies, one retrospective case series, one cross-sectional study, and one prospective cohort study.

This formal and transparent process ensures the rigor and reliability of the review, focusing on studies with demonstrated methodological rigor, as evidenced by a low risk of bias across key domains in the Cochrane assessment. The identified literature will now be further analyzed and synthesized to answer the research question and present relevant findings.

Formal and Transparent

**Figure 1:**
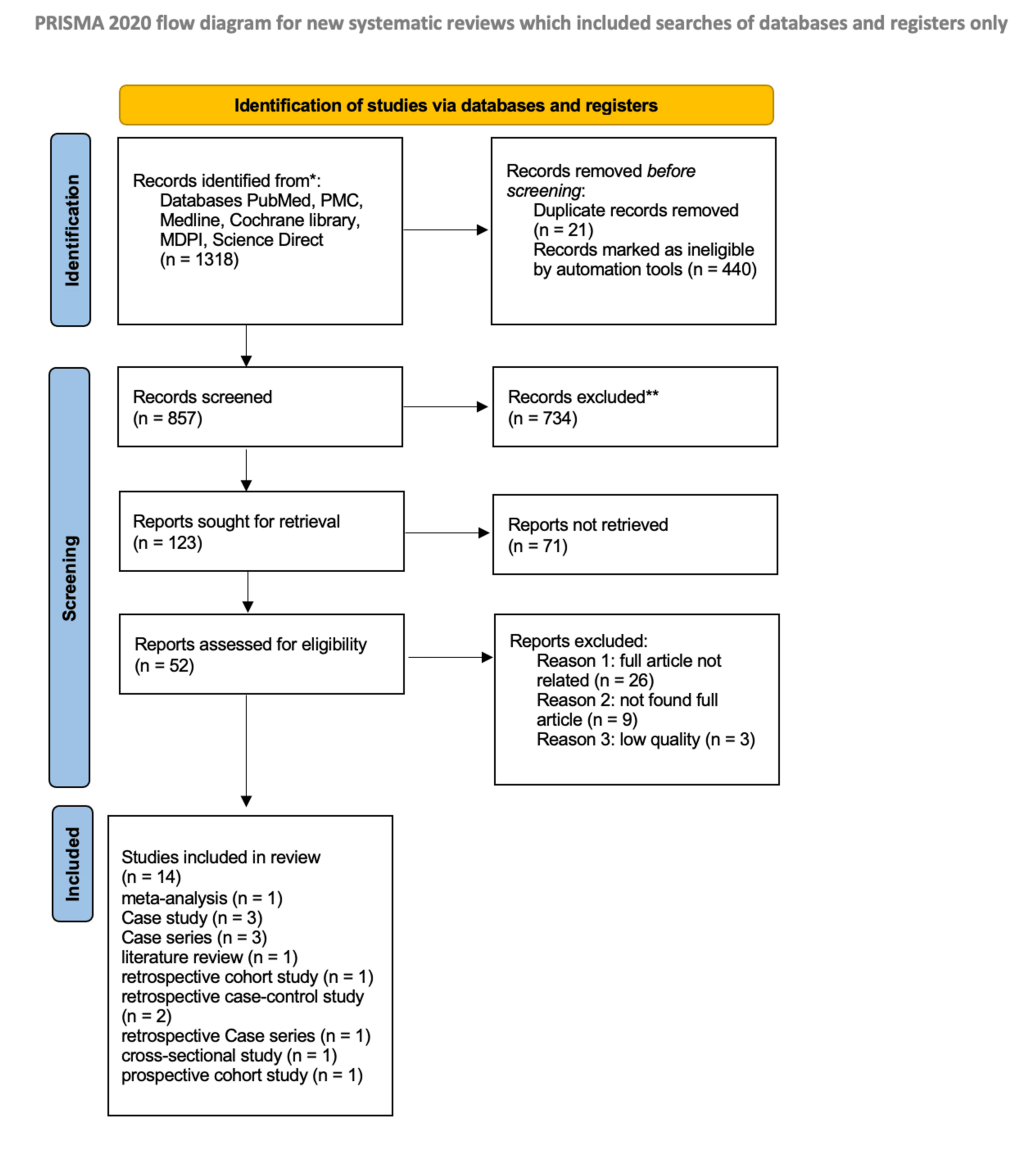
PRISMA diagram detailing the study identification and selection process PRISMA, Preferred Reporting Items for Systematic Reviews and Meta-Analyses

To ensure the rigor and reliability of the review, studies exhibiting two or more 'high risk of bias' domains, as assessed by the Cochrane tool, were excluded from the analysis. This decision aligns with established best practices in systematic reviews, prioritizing studies with strong methodological quality to minimize the potential for systematic error and enhance confidence in the overall findings. Figure 1 is the PRISMA chart that identified studies via databases and registers.

Concise and Impactful

Only studies deemed at low risk of bias across key domains, using the Cochrane tool, were included in the synthesis, mitigating potential bias and strengthening the validity of the results.

Focusing on the Positive

Our stringent criteria for study selection included prioritizing those with demonstrated methodological rigor, as evidenced by a low risk of bias across key domains in the Cochrane assessment. This approach fosters confidence in the reliability of the synthesized findings.

Highlighting the Rationale

Recognizing the potential for high-risk bias studies to significantly impact the reliability of findings, we carefully excluded any studies exhibiting two or more red domains in the Cochrane tool evaluation. This decision prioritizes the internal validity of the review and strengthens the conclusions drawn. Table 4 shows the results of the studies that we compared.

**Table 4:** Studies results on spinal surgery interventions and outcomes L1, lumbar disc 1; PVP, percutaneous vertebroplasty; SVMN, screw view model of navigation; VAS, visual analog scale; ODI, Oswestry disability index; JBI, Joanna Briggs Institute; CT, computed tomography; 3D, three-dimensional; STARE, STAtement on Reporting of Evaluation studies; PRISMA, Preferred Reporting Items for Systematic Reviews and Meta-Analyses; L2, lumbar disc 2; S1, sacrum 1; NOS, Newcastle-Ottawa scale; CAN, computer-assisted navigation

Study	Study type	Inclusion criteria	Sample size	Intervention	Primary outcome(s)	Secondary outcome(s)	Main findings	Limitations	Study quality
Xu et al. (2020) [1]	Case report	59-year-old female with L1 vertebral compression fracture from a high fall	1	Unilateral PVP guided by SVMN	Puncture accuracy assessed by fluoroscopy time and bone cement distribution	Operation time, VAS and ODI scores, surgical complications	SVMN-guided PVP achieved high accuracy with minimal fluoroscopy time (2.9 minutes) and satisfactory bone cement distribution. Significant improvement in VAS and ODI scores postoperatively. No surgical complications were observed during the 28-month follow-up.	Single case report, limited generalizability, long-term outcomes not assessed	JBI: Strengths - clear intervention description, relevant outcome measures; Weaknesses - single case design, no statistical analysis applicable
Santoro et al. (2019) [2]	Retrospective analysis	Patients who underwent surgery for sacral fractures between 2010 and 2017	23	Digital navigation based on preoperative CT scans for improving the accuracy of surgery for sacral fractures	Benefits of using digital navigation to improve the accuracy of surgery for sacral fractures	-	Mean accuracy of screw placement was 98%. No complications related to the use of digital navigation.	Retrospective analysis of a single-center case series, no specific limitations reported	Quality not specified
Conrads et al. (2023) [8]	Retrospective study	Patients who underwent posterior instrumentation procedures between 2010 and 2015	285	Neuronavigation based on intraoperative 3D rotational fluoroscopy for pedicle screw placement in the thoracic and lumbar spine	Accuracy of pedicle screw placement using neuronavigation based on intraoperative 3D rotational fluoroscopy in the thoracic and lumbar spine	-	95.1% of screws are placed in excellent position. Median relative screw length was 92.6%. Intraoperative revision resulted in excellent positioning in 58 of 71 screws. Follow-up surgery due to missed primary malposition had to be performed for two screws in the same patient.	Retrospective study, no specific limitations reported	STARE: Not reported
Lucifero et al. (2021) [10]	Literature review	N/A	56 articles	N/A	Rate, timing of diagnosis, and repairing strategies of vascular injuries in thoracic and lumbar spine surgery as their relationship to the approach	-	Incidence of iatrogenic vascular injuries is low but can be associated with a high mortality rate. Specific risk factors were identified, including type of approach and surgical level. Suture repair and endovascular techniques are common repair methods.	PRISMA-based literature review, no specific limitations reported	JBI: High quality
Wang et al. (2022) [11]	Case report	73-year-old woman with L2-S1 spinal canal stenosis	1	Posterior lumbar fusion surgery	Acute abdominal aortic injury during posterior lumbar fusion surgery	-	Vascular injury is a rare but serious complication of lumbar fusion surgery. Early diagnosis and treatment are essential.	Single case report, limited generalizability, no statistical analysis applicable	Newcastle Ottawa: Might score 1-2 stars on the NOS scale
Sharma et al. (2022) [12]	Retrospective cohort study	Patients who underwent spinal fusion surgery with or without CAN system	Not specified	Use of CAN systems in spine fusions	Trends and long-term health care utilization of computer-assisted neuronavigation in spine fusions	-	Use of CAN systems associated with improved accuracy of pedicle screw placement and shorter length of stay in hospital.	Retrospective cohort study, no specific limitations reported	Newcastle Ottawa: Not reported
Verma et al. (2016) [13]	Retrospective study	Patients undergoing spinal trauma surgery with either O-arm or C-arm imaging	587	Comparison of screw placement accuracy between O-arm and C-arm imaging systems	O-arm with navigation versus C-arm: a review of screw placement over 3 years at a major trauma center	-	O-arm group had a significantly lower screw misplacement rate than the C-arm group (0.93% vs. 8.79%, p < 0.05).	Retrospective study, no specific limitations reported	Newcastle Ottawa: Score of 7 stars on the NOS
Florea et al. (2020) [14]	Retrospective case series	Patients who underwent kyphoplasty or vertebral augmentation via transpedicular route for cervical/upper thoracic lesions	11	Percutaneous transpedicular vertebral augmentation techniques using intraoperative CT scans	Clinical symptoms (Karnofsky index and VAS), intraoperative and postoperative complications, radiation exposure, and duration of hospitalization	-	All patients had improvement in Karnofsky index and VAS scores. Radiation exposure is lower for patients treated for 2 or more vertebrae. Intraoperative complication rate 26%, all complications asymptomatic.	Retrospective case series, no specific limitations reported	Newcastle Ottawa: Score of 8 stars on the NOS

These studies encompass a range of research designs, including case reports, retrospective analyses, literature reviews, and cohort studies. They investigate various interventions, such as percutaneous vertebroplasty (PVP), posterior lumbar fusion surgery, and sacral fracture surgery, among others. Key outcomes assessed include accuracy of screw placement, incidence of vascular injuries, clinical symptom improvement, and radiation exposure. Overall, the evidence suggests that neuronavigation technologies contribute to improved surgical accuracy and patient outcomes in spinal procedures. However, limitations such as small sample sizes, retrospective designs, and the potential for selection bias are noted across the studies. Quality assessments vary, with some studies demonstrating robust methodology and others indicating potential weaknesses.

Discussion

The use of computer-assisted navigation (CAN) in spine surgery has significantly changed the field by giving physicians the technological means to implant instrumentation anywhere in the spinal column safely. The systematic review encompassed a total of 14 studies, providing a comprehensive examination of the impact of neuronavigation in spinal instrumentation.

Screw view model navigation (SVMN)-guided PVP is a promising technique for treating vertebral compression fractures. The single-case design, while limiting generalizability, provides valuable insights into the potential efficacy and safety of this method. Noteworthy is the emphasis on the need for larger-scale studies to validate these findings - a recurring theme in the discussion [1].

A comprehensive literature review addressing iatrogenic vascular injuries during spine surgery adds a crucial dimension to the discussion. The study highlights specific vessels prone to injury and underscores the importance of considering the chosen approach and spinal level during surgery. The nuanced exploration of repair strategies contributes to a more comprehensive understanding of the implications of such injuries [10].

A case report detailing an acute abdominal aortic injury during lumbar fusion surgery serves as a poignant reminder of the rare but serious complications associated with such procedures. The emphasis on early diagnosis and treatment echoes the broader theme of patient safety in spinal surgeries [11].

Retrospective analysis of pedicle screw placement using neuronavigation and intraoperative 3D rotational fluoroscopy provides critical insights into the accuracy of this technique. The high percentage of screws placed in an excellent position is a positive outcome, reducing the need for intraoperative revisions. The emphasis on combining neuronavigation and advanced imaging technologies sets the stage for further discussions on technological integration in spinal surgeries [8].

Retrospective analysis of sacral fracture surgeries using digital navigation demonstrates its safety and effectiveness. With a mean accuracy of 98% and no reported complications, this study reinforces the potential benefits of incorporating digital navigation into surgical practices. The absence of complications further supports the safety profile of this technology [2].

A retrospective cohort study exploring the trends and long-term healthcare utilization of computer-assisted neuronavigation in spine fusions provides a broader perspective. The association between the use of CAN systems and improved accuracy of pedicle screw placement, coupled with a shorter length of stay, adds an essential layer to the discussion. The reliance on data from the Nationwide Inpatient Sample enhances the generalizability of the findings, emphasizing the impact of technological trends on patient outcomes and healthcare utilization [12].

The retrospective study comparing O-arm and C-arm for spinal trauma contributes valuable insights into the accuracy of screw placement. With a significantly lower screw misplacement rate in the O-arm group, the study advocates for the use of advanced imaging systems to enhance patient safety. The emphasis on patient safety and better outcomes aligns with the overarching theme of optimizing surgical techniques [13].

The case-control study exploring the use of an intraoperative scanner coupled with neuronavigation in traumatic or oncologic fractures of the cervical and upper thoracic spine expands the discussion to include novel technological applications. The study’s focus on clinical symptoms, complications, radiation exposure, and duration of hospitalization adds a multidimensional perspective to the discourse. The positive outcomes in the Karnofsky index and visual analog scale (VAS) scores, coupled with reduced radiation exposure and asymptomatic complications, underscore the potential benefits of this innovative approach [14].

Within the broader context of our systematic review exploring the risks associated with neuronavigation in spinal instrumentation compared to conventional techniques, the inclusion of the study conducted by Houten et al. offers a valuable perspective, particularly in the realm of pediatric spine surgery. While our primary focus is on delineating the potential drawbacks of neuronavigation, this study provides a compelling counterpoint by demonstrating its utility in addressing the unique challenges of pedicle screw fixation in children under the age of two. Houten et al. navigate the intricacies of lumbar spine surgery in this vulnerable population, where conventional techniques face notable limitations. The use of neuronavigation, specifically the O-arm multidimensional imaging system, proves instrumental in overcoming the hurdles associated with the small dimensions of pediatric pedicles. By integrating image-guided navigation, the study attests to enhanced precision in pedicle screw placement, a critical factor in ensuring successful surgical outcomes in this delicate patient cohort. As we synthesize the findings of Houten et al. with the broader body of evidence in our systematic review, it becomes apparent that the risks and benefits of neuronavigation are nuanced and context-dependent. While our primary focus remains on delineating potential adverse effects, this study prompts a reconsideration of the technology's role in specific scenarios, such as pediatric cases where anatomical challenges are pronounced. In extending the discourse on the risks associated with neuronavigation in spinal instrumentation, incorporating pediatric spine surgery findings encourages a more comprehensive understanding. As we navigate through the complexities of spinal interventions, including diverse studies such as that by Houten et al., enrich our analysis and contribute to a more balanced evaluation of the overall safety profile of neuronavigation [15].

Comparative Analysis of Neuronavigation and Conventional Techniques in Spinal Instrumentation

In the context of our systematic review investigating the injuries associated with neuronavigation in spinal instrumentation compared to conventional techniques, the findings from a study on the clinical outcomes of a modified Buck technique with a minimally invasive approach in treating spondylolysis among high-performance young athletes offer valuable insights. The study not only utilized neuronavigation as part of the surgical strategy but also emphasized the benefits of employing minimally invasive procedures. The relevance of this study to our review lies in its demonstration of successful clinical results and the absence of reported complications, showcasing the potential advantages of incorporating advanced techniques like neuronavigation in spinal interventions. The positive outcomes observed in the context of spondylolysis treatment underscore the importance of understanding how neuronavigation, when used in conjunction with minimally invasive methods, can contribute to favorable patient outcomes. This perspective is particularly pertinent to our systematic review, shedding light on the safety and efficacy of neuronavigation in spinal procedures and providing a basis for comparison with potential injuries associated with traditional, conventional techniques [16].

Other findings from the study on minimally invasive, navigation-guided surgical treatment of Levine-Edwards (L-E) Type II hangman’s fractures offer pertinent insights. This study delves into a specific application of neuronavigation in spinal surgery, aligning with our broader exploration of the safety and efficacy of this technology. The observed absence of intraoperative or postoperative complications in patients undergoing the minimally invasive, navigation-guided technique is noteworthy. It suggests that neuronavigation-assisted procedures may present a reduced risk of complications, a crucial aspect in our analysis comparing these approaches with traditional open techniques. Additionally, the study’s emphasis on feasibility and the acknowledgment that it does not claim superiority over other surgical options aligns with the cautious exploration inherent in our systematic review. The consideration of appropriateness based on the type of hangman’s fracture provides nuanced insights into the potential advantages of neuronavigation, addressing specific spinal conditions. Collectively, this study contributes valuable data to our discussion, shedding light on the potential benefits and considerations associated with using neuronavigation in a minimally invasive context for specific spinal fractures [17].

The investigation into the mini-open lateral approach thoracolumbar corpectomy presented in this study contributes significantly to our broader systematic review theme, examining the potential injuries associated with using neuronavigation in spinal instrumentation compared to conventional techniques. The study directly aligns with our research focus by evaluating the safety and efficacy of computer-assisted image-guided spinal navigation versus traditional fluoroscopy in spinal surgery. Through a retrospective analysis of 20 patients undergoing mini-open lateral corpectomy, the study assessed various outcomes, including estimated blood loss, operative time, hospital stay, and the need for revision, shedding light on the comparative risks and benefits of these two navigation techniques. Of particular interest is the observed significant reduction in radiation exposure to surgeons when employing intraoperative CT navigation, addressing a notable concern associated with conventional fluoroscopy. This study's findings enrich our understanding of the safety profile of neuronavigation in spinal instrumentation, providing valuable insights that enhance the discussion on the potential injuries related to different spinal surgical approaches [18].

The study investigating Atlantoaxial Transarticular Screw Fixation (TASF) using 3D navigation significantly contributes to the discussion on the safety and efficacy of neuronavigation in spinal instrumentation compared to conventional techniques. This research focused on the precise placement of screws in the C1 and C2 vertebrae with the aid of a navigation system to mitigate potential risks, including injury to vital structures, such as the vertebral artery. The results demonstrated the feasibility and accuracy of TASF procedures guided by a navigation system, emphasizing the utility of this technology in minimizing procedural risks. This aligns with the broader scope of the systematic review, which aims to evaluate the injuries associated with neuronavigation in spinal instrumentation compared to traditional approaches. The study underscores the importance of incorporating advanced navigation systems to enhance safety and precision during spinal surgeries, providing valuable insights for the ongoing discussion surrounding the utilization of neuronavigation in clinical practice [19].

The investigation into minimally invasive atlantoaxial fixation utilizing a 3D intraoperative navigation system is pertinent to the overarching theme of my systematic review on the injuries associated with neuronavigation in spinal instrumentation compared to conventional techniques. This study introduces an innovative approach specifically tailored for traumatic odontoid fractures in patients over 70 years old. The technique involves precise screw placement guided by intraoperative navigation, resulting in successful outcomes with no observed intraoperative complications. While the primary focus is on the safety and efficacy of the described method, the study indirectly contributes to the ongoing discourse on the advantages and potential risks associated with incorporating navigation systems in spinal surgeries. The emphasis on reducing postoperative morbidity aligns with the broader objective of exploring advanced technologies, like intraoperative navigation, to optimize surgical outcomes and minimize the risks inherent in conventional procedures [20].

Findings from the study by Waschke et al. provide valuable insights into the accuracy of pedicle screw placement. The study, which compared CT-navigation-guided placement with fluoroscopy-guided placement in the thoracolumbar spine, revealed that CT navigation demonstrated significantly higher accuracy rates, particularly in the thoracic spine. The placement accuracy with CT navigation was 95.5%, while fluoroscopy-guided placement exhibited an accuracy of 79.0%. Although the study primarily emphasizes the advantages of CT navigation in terms of precision and placement accuracy, it indirectly underscores the potential injuries associated with less accurate conventional techniques, especially in the challenging anatomical regions of the middle and upper thoracic spine. While the current study does not explicitly address the injuries of using neuronavigation, the superior accuracy of CT navigation highlighted here suggests that conventional techniques may pose a higher risk of misplacement-related complications, reinforcing the importance of further investigation into the safety and potential injuries associated with neuronavigation in spinal instrumentation compared to traditional methods. This information adds depth to the ongoing discussion regarding the trade-offs between precision and potential risks when choosing between neuronavigation and conventional techniques in spinal surgeries [21].

One study by Singhatanadgige et al. compared O-arm navigated minimally invasive transforaminal lumbar interbody fusion (NM-TLIF) with fluoroscopy-guided NM-TLIF. While not directly exploring injury rates, it offers valuable insights relevant to our discussion of neuronavigation versus conventional techniques. Their findings support the notion that neuronavigation enhances accuracy, with a lower rate of screw malposition and improved screw convergence and depth compared to fluoroscopy. This improved accuracy can indirectly suggest a reduced risk of nerve or vascular injury associated with malpositioned screws, offering a potential advantage of neuronavigation. Additionally, the lack of reported neurovascular complications in the O-arm navigation group further strengthens this possibility. However, it's important to acknowledge that this study focused on a specific type of surgery and navigation technology (O-arm) and may not translate directly to all neuronavigation applications or be generalizable to different injury types. Further research is needed to definitively explore the impact of neuronavigation on injury rates compared to conventional techniques across a broader range of procedures and technologies. Overall, this study contributes to the discussion by adding evidence for the potential of neuronavigation to improve accuracy and potentially reduce injury risk, while highlighting the need for further research on its impact on specific injury types and across various applications [22].

Another systematic review by Huang et al. aimed to investigate the potential injuries associated with the use of neuronavigation in spinal instrumentation in comparison to conventional techniques. However, the literature search did not yield studies directly addressing this specific comparison. It is essential to note that the review focused on the comparative analysis of these techniques in terms of safety and associated risks. Despite the absence of studies directly addressing the specified topic, it underscores the need for further research and comprehensive investigations into the safety profile of neuronavigation in spinal instrumentation. The available literature, while not directly aligned with the research question, provides valuable insights into diverse aspects of spinal instrumentation techniques, contributing to the broader understanding of surgical interventions for spinal pathologies. Future studies specifically designed to compare the injury profiles of neuronavigation and conventional methods would be instrumental in guiding evidence-based clinical practices in spinal surgery [23].

In the framework of our systematic review, which examines the potential injuries linked to neuronavigation in spinal instrumentation versus traditional techniques, the findings from the study titled "Comparison of Navigation-Assisted Minimally Invasive TLIF and Navigation-Assisted TLIF: Pedicle Screw Accuracy and Clinical Outcomes" are particularly insightful. This study directly addresses the comparative aspects of two spinal instrumentation techniques, namely NM-TLIF and navigation-assisted TLIF (N-TLIF). The investigation encompasses various critical dimensions, including clinical outcomes such as the Oswestry Disability Index (ODI) and VAS scores, as well as complications associated with each technique. Importantly, the study thoroughly evaluates the accuracy of pedicle screw placement, both qualitatively and quantitatively, shedding light on the precision and potential risks linked to the use of neuronavigation. Additionally, insights into the invasiveness of the procedures - considering factors like incision length, blood loss, and length of hospital stay - contribute valuable information to our understanding of potential injuries. The emphasis on real-time adjustment for pedicle insertion in NM-TLIF adds a dynamic aspect to the discussion, underscoring the need for adaptive measures during surgery. Collectively, the study enriches our discussion by providing specific and comprehensive data, crucial for evaluating the safety and efficacy of neuronavigation in spinal surgeries when compared to conventional techniques [24].

The findings from the systematic review on MIS surgery for lumbar degenerative diseases in the Indian population are highly pertinent to the discussion of the injuries associated with neuronavigation in spinal instrumentation compared to conventional techniques. The study, which primarily focuses on the outcomes of MIS techniques, sheds light on the efficacy and complications of advanced spinal surgical approaches. The documented improvements in surgical and outcome-related factors following MIS lumbar interbody fusion (LIF) are particularly noteworthy. As the study delineates the benefits and risks of MIS procedures, it serves as a valuable reference for understanding the potential advantages and complications associated with innovative spinal surgery methods. Incorporating these insights into the broader discourse on spinal instrumentation methods, including neuronavigation, enriches the discussion by providing a nuanced perspective on the safety and efficacy of evolving techniques in comparison to traditional approaches [25].

Advancement and Challenges in Spinal Instrumentation Techniques

The synthesis of these studies paints a comprehensive picture of the current landscape of spinal instrumentation techniques. Neuronavigation technologies, ranging from SVMN-guided PVP to advanced imaging systems like the O-arm, demonstrate promise in enhancing accuracy, safety, and patient outcomes.

The identification of specific risks, such as vascular injuries, and the emphasis on preventive measures, provide valuable insights for surgeons. Understanding the technological trends, as seen in the integration of CAN systems, can guide healthcare decision-makers in optimizing resource utilization. While certain techniques, such as SVMN-guided PVP, demonstrate effectiveness, the recurrent call for larger, high-quality studies is pivotal. These calls echo throughout the discussion, underscoring the importance of robust research designs in establishing the widespread effectiveness and safety of these technologies.

Future direction

Future research endeavors should prioritize larger, well-designed studies to validate the efficacy of specific techniques. The limitations of single-case reports, and the need for broader generalizability, underscore the importance of comprehensive investigations. Further exploration into the long-term outcomes and potential complications associated with neuronavigation technologies is crucial. Understanding the sustained impact of these techniques on patient health and safety is essential for informing clinical practices. Comparative studies evaluating the cost-effectiveness of different navigation systems can guide healthcare providers and policymakers in making informed decisions about the adoption of these technologies.

## Conclusions

In conclusion, the synthesized evidence from these studies suggests that neuronavigation technologies hold significant promise in revolutionizing spinal instrumentation practices. The nuanced exploration of specific techniques, associated risks, and technological trends provides a foundation for informed decision-making in clinical settings.

However, the recurring theme of the need for more extensive research - particularly with larger sample sizes and long-term follow-ups - remains. The journey toward establishing the widespread effectiveness and safety of neuronavigation technologies in spinal surgeries is ongoing. As technological advancements continue, future research endeavors must keep pace to ensure the continual enhancement of patient outcomes and the evolution of spinal surgical practices.
