# Correction: Neuro Navigation Versus Conventional Spinal Techniques in Analyzing Nerve Injury and Anatomical Accuracy: A Systematic Review

**DOI:** 10.7759/cureus.c314

**Published:** 2025-09-24

**Authors:** Omar A Mahroq, Shakirat Ganiyu, Rithish Nimmagadda, Vemparala Priyatha, Bushra Firdous Shaik, Excel O Ernest-Okonofua, Safeera Khan

**Affiliations:** 1 Orthopedic Surgery, California Institute of Behavioral Neurosciences and Psychology, Fairfield, USA; 2 Internal Medicine, California Institute of Behavioral Neurosciences and Psychology, Fairfield, USA; 3 Family Medicine, California Institute of Behavioral Neurosciences and Psychology, Fairfield, USA

This article has been corrected to fix the following typos in the article text and PRISMA diagram:

- Eligibility Assessment paragraph: 'Reports excluded due to low quality of study design' has been corrected from two to three
- PRISMA diagram: 'Records identified' corrected from 1,297 to 1,318

